# Supplementary material for: Subcortical volume and white matter integrity abnormalities in major depressive disorder: findings from UK Biobank imaging data
Source: Sci Rep. 2017 Jul 17;7:5547. doi: 10.1038/s41598-017-05507-6 (PMC5514104; doi:10.1038/s41598-017-05507-6)
Supplement: Supplementary file 1 — Supplementary materials [file 41598_2017_5507_MOESM1_ESM.doc]

**Supplementary Materials**

**Subcortical volume and white matter integrity in Major Depressive Disorder (MDD): findings from UK Biobank imaging data**

**Method**

**MRI acquisition**

MRI data were acquired using a Siemens Skyra 3T scanner running VD13A SP4, with a Siemens 32-channel RF receive head coil (<https://www.healthcare.siemens.com/magnetic-resonance-imaging>). The sequence for the T1-weighted data was a standard 3D MPRAGE scan (Resolution = 1×1×1 mm, FoV = 208×256×256 matrix, TR = 2000ms, TE = 2.01ms, Orientation = sagittal, in-plane acceleration = 2, Filter = prescan-normalise). The overall duration of T1-weighted scanning was 5 minutes. For the DTI data, the diffusion preparation was a standard (“monopolar”) Stejskal-Tanner pulse sequence (Resolution = 2×2×2 mm, FoV = 104×104×72 matrix, TR = 3600ms, TE = 92.00ms, SE-EPI with x3 multislice acceleration, in-plane acceleration = off, fat saturation = on). Ten baseline volumes were collected (b = 0 s/mm^2^), with 50 b=1000 s/mm^2^ and 50 b=2000s/mm^2^. The overall duration was 7 minutes.

**MRI preprocessing**

The MRI preprocessing of both T1-weighted and DTI data were run by UK Biobank (<https://ww5.aievolution.com/hbm1601/index.cfm?do=abs.viewAbs&abs=3664>). Images were preprocessed and analysed with the FMRIB Software Library (FSL) (<http://www.fmrib.ox.ac.uk/fsl>). The IDPs from UK Biobank was released in September, 2016, which covered more than 8000 participants.

To prepare the T1-weighted volumes for standard pre-processing procedures, the face area was removed to maintain anonymity. Following this, gradient distortion correction was applied for the whole image using BET (Brain Extraction Tool) ^1^ and FLIRT (FMRIB's Linear Image Registration Tool) ^2,3^. The brain was non-linearly warped to the MNI152 "nonlinear 6th generation" standard-space T1-weighted volume template, and the brain area of the images was then extracted using FNIRT (FMRIB's Nonlinear Image Registration Tool) ^4^ for segmentation. Segmentation of brain was conducted in two steps: firstly, a tissue-type segmentation using FAST (FMRIB's Automated Segmentation Tool) ^5^ was applied to extract cerebrospinal fluid, grey matter and white matter; then subcortical structures are extracted using FIRST (FMRIB's Integrated Registration and Segmentation Tool) ^6^. The volumes of ICV, thalamus, putamen, pallidum, hippocampus, caudate, brain stem, amygdala and accumbens were calculated for further analysis.

DTI data was initially corrected by the Eddy tool for eddy currents, head motion and outlier-slices ^7^, and the following gradient distortion correction was applied in the same way as it is applied on T1-weighted volumes. The corrected b=1000 s/mm^2^ shell was then used for modeling whole brain water diffusivity biomarkers using DTIFIT, thereby creating the FA (fractional anisotropy) maps.

The DTI data we used was processed by UK biobank using a probabilistic tractography based method. FA maps were initially warped to standard space, and then the BEDPOSTx tool (Bayesian Estimation of Diffusion Parameters Obtained using Sampling Techniques) was used to generate the fibres derived from major anatomical seeds (<http://fsl.fmrib.ox.ac.uk/fsl/fslwiki/FDT/UserGuide>). This maps 27 major tracts (12 bilateral tracts in both hemispheres and 3 tracts that went across brain) by utilizing the standard-space start/stop ROI masks defined by AutoPtx ^8^.

**Statistical methods**

We used lme function in nlme package of R ^9^ to conduct repeated-effect linear model on the structures with bilateral measures, as hemisphere was a within-subject variable, whereas all other covariates and the variable of interest are between-subject variables. The general linear model of unilateral structures was conducted using the default glm function of R. Choices of covariates were based on the recent meta-analytic studies on big samples of psychiatric illnesses ^10,11^.

**Participants**

The acquisition and preprocessing were conducted by UK biobank. 5724 participants finished T1 image acquisition and the scans were preprocessed, while 4941 participants’ DTI images were acquired and preprocessed. After the outliers were excluded, there were 5403 with T1 images and 4594 with DTI images. Outlier exclusion was conducted within the overall sample with according imaging data available, therefore this step of exclusion is unbiased against the final samples which were consisted of only MDD cases and healthy controls. For transparency, the results of the main models that tested the effect of MDD definitions, with or without excluding outliers, were both presented in the tables below (Table S4, S5, S7, S8). Then the participants that had a diagnosis of Parkinson’s disease, bipolar disorder, multiple personality disorder, schizophrenia, autism or intellectual disability were also excluded.

After the applying the filters described above, cases and controls for MDD definitions were chosen according to their self-reported depressive symptoms and hospital admission history (see below in Method, MDD definitions). Details of the sample and exclusions were listed in Table S3.

**MDD definitions**

The putative MDD category summarized by Smith et al. was based on depressive symptoms and hospital admission history reported by participants. Self-report symptoms included whether they had ever been depressed or had anhedonia, whether they experienced a depressive period of over two weeks, and how many depressive episodes they had. Hospital admission history was also self-reported by answering whether they have seen a GP or psychiatrist for nerves, anxiety, tension or depression. As described in Figure S4, people were categorized into four groups: single episode major depression, recurrent major depression (moderate), recurrent major depression (severe) and absent of depression. These MDD categories were tested over phenotypes of lifestyle, demographics, social states, overall health condition and emotion disorder related personality. The results showed similar patterns with clinical ascertained samples ^12^. The tests were conducted in the sample of 172,751 participants of UK Biobank. Though participants who had imaging assessments were recruited within this pretested sample, we compared neuroticism level between cases and controls in the current, smaller sample to validate the MDD definitions we used as below.

In addition to their MDD categories, we added another category as unspecified group. They reported depressive symptoms or relative hospital admission history, but did not meet the criteria to be categorized as MDD. They either reported of having had at least two weeks duration of low mood or anhedonia, and at least 2 episodes of depression, but had not seen a GP/psychiatrist; or reported of having had seen a GP/psychiatrist and had at least two weeks duration of low mood or anhedonia, but didn't know episodes or duration.

For the principal definition of MDD, cases included recurrent and single-episode MDD, and controls included only those who were identified of being depression absent. For the definition of recurrent MDD, cases were only recurrent MDD, whilst the controls included the rest of the categories, which included single-episode MDD, depression absent participants and participants who weren’t identified as MDD but self-reported of having had depressive symptoms or had hospital admission history of seeing a GP or a psychiatrist for nerves, anxiety, depression. See Figure S4. The participants who did not respond to any of the questions used as criteria for categorization were excluded.

In the sample with T1-weighted data, MDD cases have significantly higher neuroticism level in both principal and recurrent MDD definition, β = 0.678, p < 2e-16; β = 0.555, p = 2e-16 respectively. The differences remained the same if age, age^2^, sex were set as covariates, β = 0.600, p < 2e-16; β = 0.480, p = 2.84e-13. Comparisons were again conducted within the sample with DTI data. They similarly showed that cases were more neurotic than controls in both definitions, with or without controlling sex and age, βs = 0.550~0.717, ps < 7.36e-16. The above neuroticism scores were calculated using the same method in the prevalence study by Smith et al ^12^.

We tested separately in both MDD definitions on group differences of gender, age and level of education between cases and controls. Level of education was coded as below: A levels/AS levels = 6, O levels/GCSEs = 5, CSEs = 4, NVQ or HND or HNC = 3, Other qualifications =2, No respond/refuse to answer = 1. Gender differences were significant in both definitions (***χ***^2^_probable_ = 35.43, df = 1, p_probable_ = 2.64e-9; ***χ***^2^_recurrent_ = 4.74, df = 1, p_recurrent_ = 0.030 respectively for probable and recurrent definition for T1-weighted sample. ***χ***^2^ _probable_ = 30.90, df = 1, p_probable_ = 2.72e-8; ***χ***^2^ _recurrent_ = 12.90, df = 1, p_recurrent_ = 3.29e-4 for DTI sample). Age differences were also significant (T1-weighted sample: β_probable_ = -0.296, p_probable_ = 1. 57e-6; β_recurrent_ = -0.242, p_recurrent_ = 2.57e-4; DTI sample: β_probable_ = -0.302, p_probable_ = 3.20e-6; β_recurrent_ = -0.278, p_recurrent_ = 6.81e-5). Difference of education level was not significant (T1-weighted sample: β_probable_ = 0.001, p_probable_ = 0.984; β_recurrent_ = 0.061, p_recurrent_ = 0.387; DTI sample: β_probable_ = 0.012, p_probable_ = 0.861; β_recurrent_ = 0.063, p_recurrent_ = 0.387), and the differences were even lower when age and sex were set as covariates (ps > 0.369). Many previous meta-analyses included only age and sex as covariates, and the recent protocol paper of UKB brain imaging phenotypes stated that sex and age could largely influence tests. The above descriptive statistics also reassured that the differences of age and sex between cases and controls were significant, while education differences were not robustly large. Therefore, we set sex, age, age^2^ and assessment centre as covariates in all the models, whereas additional model to test the effect of MDD definitions on FA values included education level and number of release as covariates was tested, and the results remained the same (Table S10).

For the findings on the PCA scores on FA, association/commissural fibres, thalamic radiations and projection fibres, we similarly checked the effect of self-declare depression. Among the DTI-data sample, there were 239 self-declare depression cases and 4349 controls. We found that self-declare depression cases showed decreased gFA (β=-0.14, p=0.026), gAF (β=-0.14, p=0.032) and gTR (β=-0.17, p=0.009). This self-declare status was collected based on a general report of non-cancer illnesses on data field 20002 of UK Biobank touchscreen-assessment data (<http://biobank.ctsu.ox.ac.uk/crystal/field.cgi?id=20002>). This question was a general question to which participants were to recall all non-cancer illnesses that they had, and no hospital admission record was considered. Therefore was only used in validation tests on the findings of PCA components.

**References**

1. Smith, S. M. Fast robust automated brain extraction. *Hum. Brain Mapp.* **17,** 143–155 (2002).

2. Jenkinson, M. & Smith, S. M. A global optimization method for robust affine registration of brain images. *Med. Imaging Anal.* **5,** 143–156 (2001).

3. Jenkinson, M., Bannister, P., Brady, M. & Smith, S. Improved optimization for the robust and accurate linear registration and motion correction of brain images. *Neuroimage* **17,** 825–841 (2002).

4. Andersson, J. L. R., Jenkinson, M. & Smith, S. Non-linear registration aka Spatial normalisation FMRIB Technial Report TR07JA2. *In Pract.* 22 (2007). at <http://fmrib.medsci.ox.ac.uk/analysis/techrep/tr07ja2/tr07ja2.pdf>

5. Zhang, Y., Brady, M. & Smith, S. Segmentation of brain MR images through a hidden Markov random field model and the expectation-maximization algorithm. *Med. Imaging, IEEE Trans.* **20,** 45–57 (2001).

6. Patenaude, B., Smith, S. M., Kennedy, D. N. & Jenkinson, M. A Bayesian model of shape and appearance for subcortical brain segmentation. *Neuroimage* **56,** 907–922 (2011).

7. Andersson, J. L. R. & Sotiropoulos, S. N. Non-parametric representation and prediction of single- and multi-shell diffusion-weighted MRI data using Gaussian processes. *Neuroimage* **122,** 166–176 (2015).

8. De Groot, M. *et al.* Improving alignment in Tract-based spatial statistics: Evaluation and optimization of image registration. *Neuroimage* **76,** 400–411 (2013).

9. Bliese, P. *Multilevel Modeling in R (2.2)--A Brief Introduction to R, the multilevel package and the nlme package*. (2016). at <https://cran.r-project.org/doc/contrib/Bliese_Multilevel.pdf>

10. Schmaal, L. *et al.* Subcortical brain alterations in major depressive disorder: findings from the ENIGMA Major Depressive Disorder working group. *Mol. Psychiatry* **21,** 806–812 (2016).

11. van Erp, T. G. M. *et al.* Subcortical brain volume abnormalities in 2028 individuals with schizophrenia and 2540 healthy controls via the ENIGMA consortium. *Mol. Psychiatry* **21,** 547–553 (2016).

12. Smith, D. J. *et al.* Prevalence and characteristics of probable major depression and bipolar disorder within UK biobank: cross-sectional study of 172,751 participants. *PLoS One* **8,** e75362 (2013).


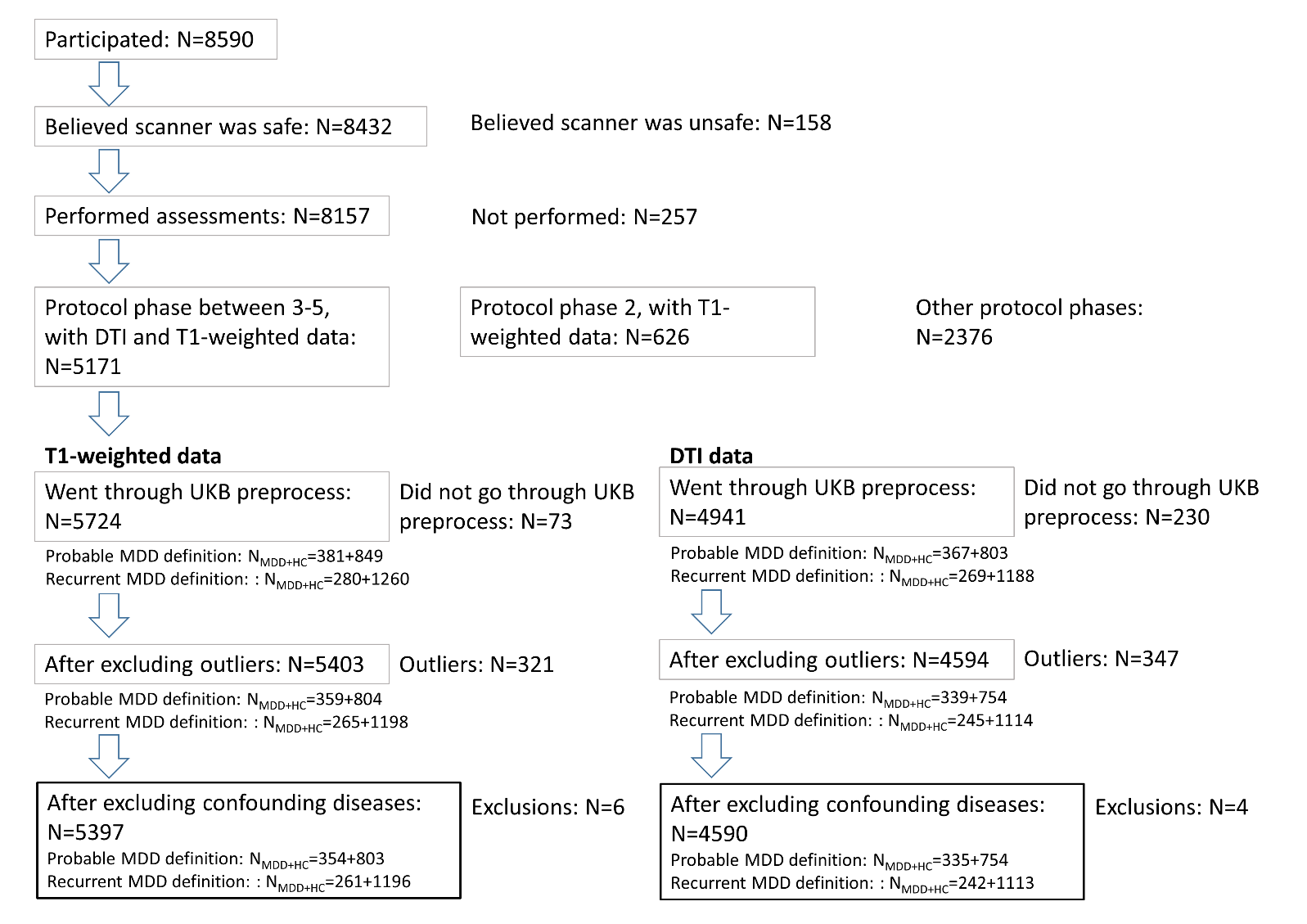


Figure S1. Sample size change after each step of exclusion. The boxes with grey outline were kept for the next step. For the steps “went through UKB preprocess” and “After excluding outliers” , number of participants with imaging data and the numbers of subjects included as a case or control in both definitions were stated separately.


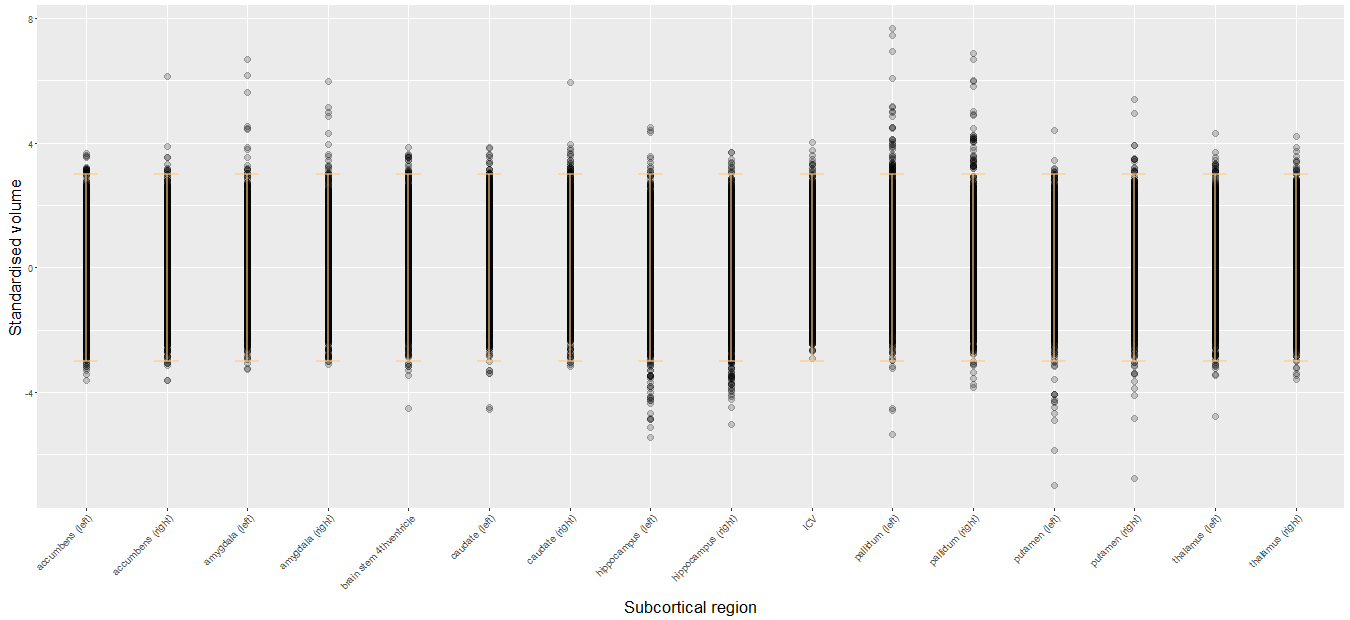


Figure S2. Standardised data of subcortical volumes. Each data point represents one person/region. The number of participants excluded as outliers was state in Figure S1. The error bars represent +/-3 standard deviation.


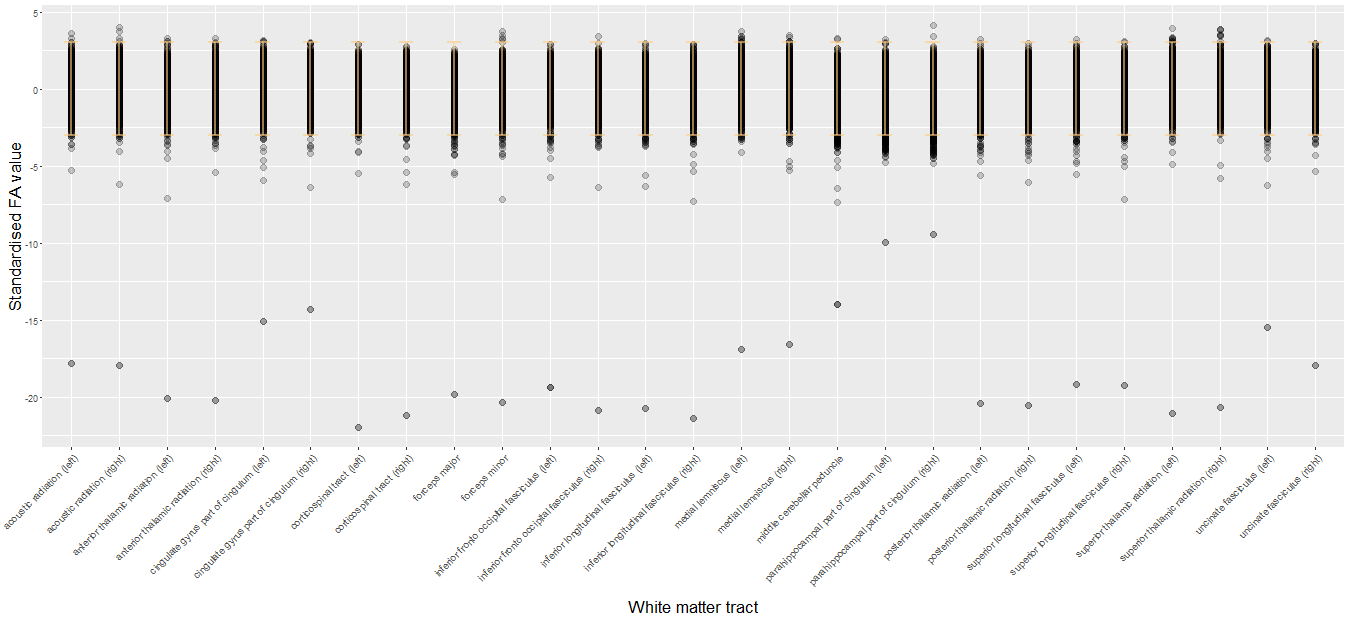


Figure S3. Standardised data of white matter integrity. Each data point represents one person/region. The number of participants excluded as outliers was state in Figure S1. The error bars represent +/-3 standard deviation.


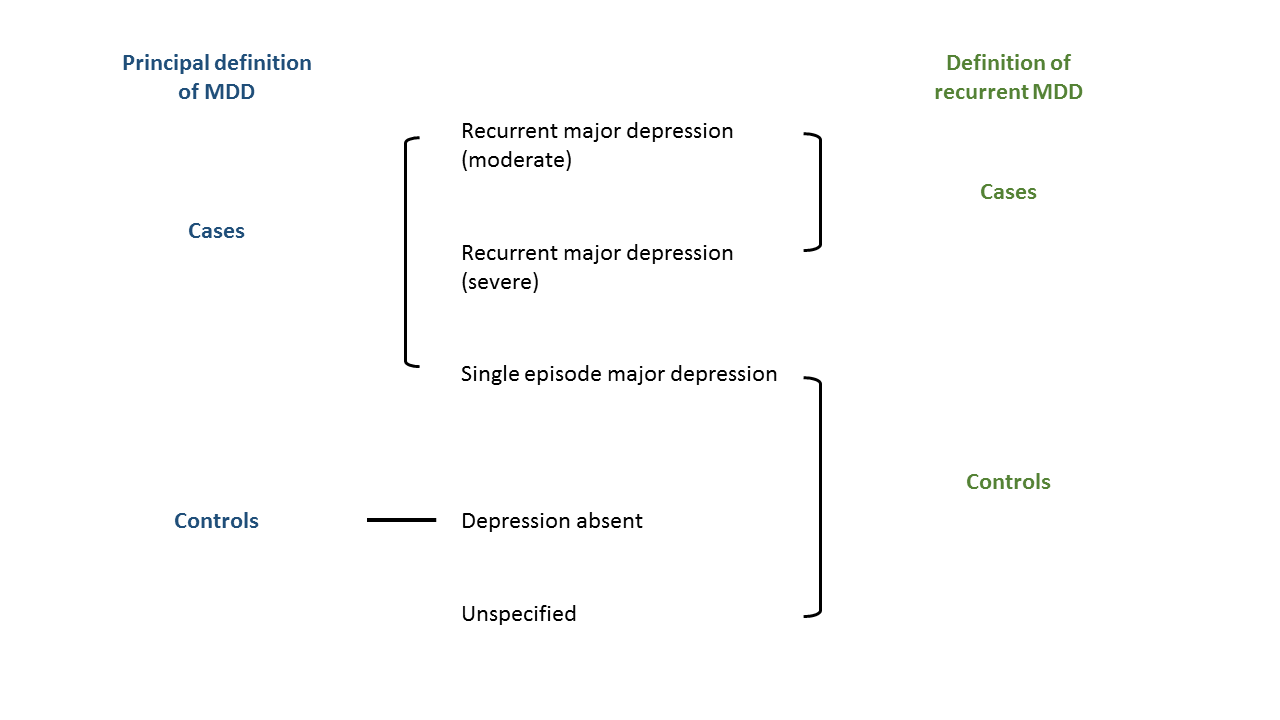


Figure S4. Principal definition of MDD and the definition of recurrent MDD. The categorization of moderate and severe recurrent major depression, single episode major depression and depression absent were summarized by Smith *et al.* (2013). An additional group of participants who self-reported of having had depressive symptoms or hospital admission history of nerves, anxiety or depression were categorized as ‘unspecified’. The principal definition of MDD compared all MDD cases with those who were depression absent, and the definition of recurrent MDD compared recurrent MDD verses single-episode MDD, participants who were depression absent and the unspecified group.

Table S1. Descriptive statistics of imaging phenotypes. The statistics were concluded from the samples with imaging data regardless of MDD definitions (see Figure S1). Briefly, the raw T1-weighted data included 5724 people, and there were 5403 remained after the QC. Raw DTI data included 4941 people, and 4594 remained after the QC.

T1-weighted data:

| **Tract** | **Raw data** | |  | **Data after QC** | |
| --- | --- | --- | --- | --- | --- |
|  | **Mean** | **SD** |  | **Mean** | **SD** |
| thalamus (left) | 7799.39 | 752.42 |  | 7781.45 | 709.95 |
| thalamus (right) | 7603.63 | 728.01 |  | 7586.65 | 689.60 |
| caudate (left) | 3396.74 | 421.10 |  | 3382.02 | 399.54 |
| caudate (right) | 3573.61 | 440.54 |  | 3555.72 | 416.01 |
| putamen (left) | 4815.74 | 604.28 |  | 4808.70 | 567.80 |
| putamen (right) | 4859.38 | 586.82 |  | 4848.13 | 552.87 |
| pallidum (left) | 1763.60 | 243.70 |  | 1751.53 | 217.47 |
| pallidum (right) | 1809.15 | 244.23 |  | 1798.13 | 218.11 |
| hippocampus (left) | 3813.23 | 474.68 |  | 3817.03 | 438.50 |
| hippocampus (right) | 3925.20 | 485.96 |  | 3926.84 | 450.72 |
| amygdala (left) | 1277.81 | 248.99 |  | 1273.88 | 237.74 |
| amygdala (right) | 1246.91 | 276.44 |  | 1243.24 | 266.44 |
| accumbens (left) | 507.55 | 120.42 |  | 506.79 | 115.44 |
| accumbens (right) | 402.64 | 111.31 |  | 402.37 | 107.82 |
| brain stem | 22857.44 | 2764.28 |  | 22772.54 | 2635.68 |
| ICV | 1203924.85 | 115196.69 |  | 1199108.04 | 110698.23 |

DTI data:

| **Tract** | **Raw data** | |  | **Data after QC** | |
| --- | --- | --- | --- | --- | --- |
|  | **Mean** | **SD** |  | **Mean** | **SD** |
| acoustic radiation (left) | 0.423 | 0.024 |  | 0.424 | 0.022 |
| acoustic radiation (right) | 0.414 | 0.023 |  | 0.415 | 0.021 |
| anterior thalamic radiation (left) | 0.401 | 0.020 |  | 0.401 | 0.017 |
| anterior thalamic radiation (right) | 0.393 | 0.019 |  | 0.394 | 0.017 |
| cingulate gyrus part of cingulum (left) | 0.537 | 0.036 |  | 0.538 | 0.033 |
| cingulate gyrus part of cingulum (right) | 0.498 | 0.035 |  | 0.499 | 0.033 |
| parahippocampal part of cingulum (left) | 0.312 | 0.031 |  | 0.315 | 0.027 |
| parahippocampal part of cingulum (right) | 0.311 | 0.033 |  | 0.314 | 0.028 |
| corticospinal tract (left) | 0.547 | 0.025 |  | 0.548 | 0.022 |
| corticospinal tract (right) | 0.541 | 0.026 |  | 0.542 | 0.022 |
| forceps major | 0.582 | 0.029 |  | 0.583 | 0.026 |
| forceps minor | 0.466 | 0.023 |  | 0.467 | 0.020 |
| inferior fronto occipital fasciculus (left) | 0.476 | 0.025 |  | 0.477 | 0.020 |
| inferior fronto occipital fasciculus (right) | 0.466 | 0.022 |  | 0.467 | 0.019 |
| inferior longitudinal fasciculus (left) | 0.461 | 0.022 |  | 0.462 | 0.019 |
| inferior longitudinal fasciculus (right) | 0.452 | 0.021 |  | 0.453 | 0.018 |
| middle cerebellar peduncle | 0.477 | 0.034 |  | 0.479 | 0.030 |
| medial lemniscus (left) | 0.418 | 0.025 |  | 0.419 | 0.023 |
| medial lemniscus (right) | 0.421 | 0.025 |  | 0.422 | 0.024 |
| posterior thalamic radiation (left) | 0.458 | 0.022 |  | 0.459 | 0.020 |
| posterior thalamic radiation (right) | 0.454 | 0.022 |  | 0.456 | 0.019 |
| superior longitudinal fasciculus (left) | 0.442 | 0.023 |  | 0.443 | 0.020 |
| superior longitudinal fasciculus (right) | 0.425 | 0.022 |  | 0.426 | 0.019 |
| superior thalamic radiation (left) | 0.423 | 0.020 |  | 0.424 | 0.018 |
| superior thalamic radiation (right) | 0.422 | 0.020 |  | 0.423 | 0.018 |
| uncinate fasciculus (left) | 0.391 | 0.025 |  | 0.392 | 0.023 |
| uncinate fasciculus (right) | 0.391 | 0.022 |  | 0.392 | 0.020 |

Table S2. Major depressive disorder criteria summarized by Smith et al. (2013)

| **Category** | Criteria |
| --- | --- |
| **Single probable episode of major depression** | Ever depressed/down for a whole week, plus at least two weeks duration, plus only one episode, plus ever seen a GP or a psychiatrist for nerves, anxiety, depression.  OR |
|  | Ever anhedonia (unenthusiasm/uninterest) for a whole week, plus at least two weeks, plus only one episode, plus ever seen a GP or a psychiatrist for nerves, anxiety, depression |
| **Probable recurrent major depression (moderate)** | Ever depressed/down for a whole week, plus at least two weeks duration, plus at least two episodes, plus ever seen a GP (but not a psychiatrist) for nerves, anxiety, depression  OR |
|  | Ever anhedonia (unenthusiasm/uninterest) for a whole week, plus at least two weeks, plus at least two episodes, plus ever seen a GP (but not a psychiatrist) for nerves, anxiety, depression |
| **Probable recurrent major depression (severe)** | Ever depressed/down for a whole week, plus at least two weeks duration, plus at least two episodes, plus ever seen a psychiatrist for nerves, anxiety, depression  OR |
|  | Ever anhedonia (unenthusiasm/uninterest) for a whole week, plus at least two weeks, plus at least two episodes, plus ever seen a psychiatrist for nerves, anxiety, depression |
| **Depression absent** | 1. Mood question answered 'no' 2. Reported symptoms but duration was too short. 3. Reported symptoms but period was below threshold. 4. Had not seen GP or psychiatrist and did not self-report depression |

Note: Participants needed to meet all four criterion to be categorized as depression absent.

Table S3. Demographic features of samples with T1-weighted and DTI data. The descriptive statistics below are summarised based on the samples that were analysed in the present study (see the final sample size in Figure S1).

Subjects with T1-weighted data:

|  | Principal MDD definition | |  | Recurrent MDD definition | |  |
| --- | --- | --- | --- | --- | --- | --- |
|  | Case | Control | N | Case | Control | N |
| Sample size | 354 | 803 | -- | 261 | 1196 | -- |
| Age (Mean±SD) | 54.97±7.38 | 57.19±7.14 | 1157 | 54.99±7.33 | 56.80±7.21 | 1457 |
| Number of Male | 123 | 433 | 1157 | 97 | 593 | 1457 |
| Proportion of Male (%) | 34.75 | 53.92 |  | 37.16 | 49.58 |  |
| Average Education level | 4.74 | 4.74 | 1157 | 4.78 | 4.69 | 1157 |
| College or University degree (%) | 45.48 | 40.72 | -- | 46.36 | 39.38 | -- |
| A levels/AS levels (%) | 13.28 | 15.82 | -- | 14.94 | 15.05 | -- |
| O levels/GCSEs (%) | 18.36 | 21.30 | -- | 16.09 | 21.57 | -- |
| CSEs (%) | 5.93 | 4.23 | -- | 6.13 | 4.68 | -- |
| NVQ or HND or HNC (%) | 5.65 | 5.48 | -- | 5.75 | 5.77 | -- |
| Other qualifications (%) | 5.65 | 4.61 | -- | 5.36 | 4.85 | -- |
| No respond/refuse to answer (%) | 5.65 | 7.85 | -- | 5.36 | 8.70 | -- |

Subjects with DTI data:

|  | Principal MDD definition | |  | Recurrent MDD definition | |  |
| --- | --- | --- | --- | --- | --- | --- |
|  | Case | Control | N | Case | Control | N |
| Sample size | 335 | 754 | -- | 242 | 1113 | -- |
| Age (Mean±SD) | 54.83±7.40 | 57.07±7.24 | 1089 | 54.63±7.34 | 56.69±7.24 | 1355 |
| Number of Male | 119 | 408 | 1089 | 91 | 563 | 1355 |
| Proportion of Male (%) | 35.52 | 54.11 |  | 37.60 | 50.58 |  |
| Average Education level | 4.74 | 4.72 | 1089 | 4.78 | 4.69 | 1355 |
| College or University degree () | 46.27 | 39.66 | -- | 47.93 | 38.90 | -- |
| A levels/AS levels () | 12.84 | 16.71 | -- | 14.05 | 15.45 | -- |
| O levels/GCSEs () | 19.10 | 21.22 | -- | 16.94 | 21.56 | -- |
| CSEs () | 5.97 | 3.98 | -- | 6.20 | 4.58 | -- |
| NVQ or HND or HNC () | 5.37 | 5.44 | -- | 5.37 | 5.84 | -- |
| Other qualifications () | 5.97 | 4.91 | -- | 5.79 | 4.67 | -- |
| No respond/refuse to answer () | 4.48 | 8.09 | -- | 3.72 | 8.98 | -- |

Table S4. The effect of MDD definition on the volumes of subcortical regions and brain matters (without excluding outliers). The same model was conducted with age, age^2^, sex and assessment centre set as covariates. Hemisphere was also set as a covariate when appropriate. Sample sizes were N_case_=381, N_control_=849 and N_case_=280, N_control_=1260 for principal and recurrent definitions respectively.

| Subcortical regions |  | **Principal definition** | | | | |  |  | **Recurrent definition** | | | | |
| --- | --- | --- | --- | --- | --- | --- | --- | --- | --- | --- | --- | --- | --- |
|  | Effect size | | Standard deviation | t value | p value | p_corrected_ |  | Effect size | | Standard deviation | t value | p value | p_corrected_ |
| **Accumbens** | 0.013 | | 0.048 | 0.277 | 0.782 | 0.879 |  | -0.012 | | 0.051 | -0.247 | 0.805 | 0.913 |
| **Amygdala** | -0.032 | | 0.049 | -0.658 | 0.511 | 0.879 |  | 0.026 | | 0.052 | 0.497 | 0.620 | 0.913 |
| **Caudate** | 0.047 | | 0.050 | 0.923 | 0.356 | 0.879 |  | 0.017 | | 0.053 | 0.329 | 0.742 | 0.913 |
| **Hippocampus** | -0.036 | | 0.049 | -0.724 | 0.469 | 0.879 |  | -0.056 | | 0.051 | -1.089 | 0.276 | 0.829 |
| **Pallidum** | 0.014 | | 0.051 | 0.277 | 0.782 | 0.879 |  | 0.006 | | 0.054 | 0.110 | 0.913 | 0.913 |
| **Putamen** | 0.020 | | 0.046 | 0.429 | 0.668 | 0.879 |  | -0.006 | | 0.047 | -0.119 | 0.905 | 0.913 |
| **Thalamus** | -0.058 | | 0.038 | -1.543 | 0.123 | 0.879 |  | -0.067 | | 0.040 | -1.695 | 0.090 | 0.813 |
| **Brain stem** | -0.007 | | 0.051 | -0.134 | 0.893 | 0.893 |  | 0.043 | | 0.054 | 0.797 | 0.425 | 0.913 |
| **ICV** | -0.058 | | 0.045 | -1.287 | 0.198 | 0.879 |  | -0.062 | | 0.048 | -1.295 | 0.196 | 0.829 |

Table S5. The interaction between MDD definition and hemisphere on the volumes of subcortical regions and brain matters. In this model, again age, age^2^, sex and assessment centre were set as covariates. MDD definition, hemisphere and the interaction between MDD definition and hemisphere were also included in the model. As brain stem and ICV were unilateral structure/measure, therefore these two measure were not tested in this model.

| Subcortical regions |  | **Principal definition** | | | | |  |  | **Recurrent definition** | | | | |
| --- | --- | --- | --- | --- | --- | --- | --- | --- | --- | --- | --- | --- | --- |
|  | Effect size | | Standard deviation | t value | p value | p_corrected_ |  | Effect size | | Standard deviation | t value | p value | p_corrected_ |
| **Accumbens** | -0.014 | | 0.056 | -0.250 | 0.803 | 0.907 |  | -0.064 | | 0.059 | -1.088 | 0.277 | 0.879 |
| **Amygdala** | 0.066 | | 0.070 | 0.936 | 0.349 | 0.907 |  | -0.011 | | 0.074 | -0.143 | 0.886 | 0.945 |
| **Caudate** | 0.011 | | 0.028 | 0.388 | 0.698 | 0.907 |  | -0.008 | | 0.030 | -0.280 | 0.779 | 0.945 |
| **Hippocampus** | 0.034 | | 0.054 | 0.633 | 0.527 | 0.907 |  | 0.004 | | 0.059 | 0.070 | 0.945 | 0.945 |
| **Pallidum** | -0.006 | | 0.048 | -0.117 | 0.907 | 0.907 |  | -0.015 | | 0.051 | -0.290 | 0.772 | 0.945 |
| **Putamen** | -0.047 | | 0.034 | -1.383 | 0.167 | 0.907 |  | -0.047 | | 0.036 | -1.312 | 0.190 | 0.879 |
| **Thalamus** | 0.015 | | 0.023 | 0.671 | 0.502 | 0.907 |  | 0.022 | | 0.024 | 0.884 | 0.377 | 0.879 |

Table S6. The effect of MDD definition on FA values of DTI tracts (gFA included as a convariate). In order to test whether the significant effect of MDD definitions remains significant when general FA change was controlled, this model included gFA score as a covariate. The method to extract gFA score was stated in the main text (Methods-Statistical methods-White matter integrity).

| DTI tracts | **Principal definition** | | | |  |  | **Recurrent definition** | | | |  |
| --- | --- | --- | --- | --- | --- | --- | --- | --- | --- | --- | --- |
|  | Effect size | Standard deviation | t value | p value | p_corrected_ |  | Effect size | Standard deviation | t value | p value | p_corrected_ |
| **Acoustic radiation** | 0.030 | 0.043 | 0.707 | 4.80E-001 | 0.849 |  | 0.006 | 0.046 | 0.136 | 8.92E-001 | 0.946 |
| **Anterior thalamic radiation** | 0.062 | 0.039 | 1.572 | 1.16E-001 | 0.372 |  | 0.056 | 0.042 | 1.349 | 1.78E-001 | 0.568 |
| **Cingulate gyrus part of cingulum** | -0.026 | 0.046 | -0.563 | 5.74E-001 | 0.849 |  | -0.008 | 0.049 | -0.173 | 8.63E-001 | 0.946 |
| **Corticospinal tract** | 0.003 | 0.051 | 0.053 | 9.58E-001 | 0.958 |  | 0.023 | 0.055 | 0.415 | 6.78E-001 | 0.946 |
| **Inferior fronto-occipital fasciculus** | 0.029 | 0.031 | 0.928 | 3.53E-001 | 0.808 |  | 0.007 | 0.032 | 0.221 | 8.25E-001 | 0.946 |
| **Inferior longitudinal fasciculus** | 0.017 | 0.032 | 0.548 | 5.84E-001 | 0.849 |  | -0.010 | 0.032 | -0.316 | 7.52E-001 | 0.946 |
| **Medial lemniscus** | 0.007 | 0.056 | 0.117 | 9.07E-001 | 0.958 |  | 0.018 | 0.058 | 0.312 | 7.55E-001 | 0.946 |
| **Parahippocampal part of cingulum** | -0.012 | 0.054 | -0.218 | 8.27E-001 | 0.958 |  | 0.010 | 0.057 | 0.171 | 8.64E-001 | 0.946 |
| **Posterior thalamic radiation** | 0.037 | 0.045 | 0.808 | 4.19E-001 | 0.839 |  | 0.013 | 0.047 | 0.265 | 7.91E-001 | 0.946 |
| **Superior longitudinal fasciculus (bilateral)** | 0.006 | 0.035 | 0.174 | 8.62E-001 | 0.958 |  | -0.021 | 0.038 | -0.566 | 5.72E-001 | 0.946 |
| **Superior longitudinal fasciculus (left)** | -0.194 | 0.066 | -2.951 | 3.23E-003 | 0.038 |  | -0.221 | 0.070 | -3.165 | 1.59E-003 | 0.025 |
| **Superior thalamic radiation** | -0.110 | 0.051 | -2.168 | 3.03E-002 | 0.162 |  | -0.077 | 0.053 | -1.442 | 1.50E-001 | 0.568 |
| **Uncinate fasciculus** | 0.013 | 0.040 | 0.330 | 7.42E-001 | 0.958 |  | -0.003 | 0.043 | -0.068 | 9.46E-001 | 0.946 |
| **Forceps major** | -0.193 | 0.068 | -2.834 | 4.69E-003 | 0.038 |  | -0.133 | 0.072 | -1.842 | 6.57E-002 | 0.350 |
| **Forceps minor** | -0.112 | 0.065 | -1.723 | 8.52E-002 | 0.341 |  | -0.159 | 0.070 | -2.266 | 2.36E-002 | 0.189 |
| **Middle cerebellar peduncle** | -0.066 | 0.064 | -1.024 | 3.06E-001 | 0.808 |  | 0.039 | 0.068 | 0.576 | 5.65E-001 | 0.946 |

Table S7. The effect of MDD definition on FA values of DTI tracts (Without excluding outliers). The same model for Table 2 was conducted, with age, age^2^, sex and assessment centre controlled and hemisphere also controlled when appropriate. Sample sizes were N_case_=367, N_control_=803 and N_case_=269, N_control_=1188 for principal and recurrent definitions respectively. The standard effect sizes of significant tracts found within the sample that outliers were excluded remained in similar trend. Significant tracts included left superior longitudinal fasciculus, forceps major and superior thalamic radiation.

| DTI tracts | **Principal definition** | | | |  |  | **Recurrent definition** | | | |  |
| --- | --- | --- | --- | --- | --- | --- | --- | --- | --- | --- | --- |
|  | Effect size | Standard deviation | t value | p value | p_corrected_ |  | Effect size | Standard deviation | t value | p value | p_corrected_ |
| **Acoustic radiation** | -0.055 | 0.052 | -1.053 | 2.92E-001 | 0.425 |  | -0.032 | 0.065 | -0.488 | 6.26E-001 | 0.807 |
| **Anterior thalamic radiation** | -0.042 | 0.055 | -0.759 | 4.48E-001 | 0.506 |  | -0.002 | 0.069 | -0.024 | 9.81E-001 | 0.981 |
| **Cingulate gyrus part of cingulum** | -0.072 | 0.054 | -1.348 | 1.78E-001 | 0.376 |  | -0.021 | 0.064 | -0.322 | 7.48E-001 | 0.854 |
| **Corticospinal tract** | -0.091 | 0.056 | -1.626 | 1.04E-001 | 0.376 |  | -0.071 | 0.061 | -1.151 | 2.50E-001 | 0.807 |
| **Inferior fronto-occipital fasciculus** | -0.038 | 0.053 | -0.715 | 4.75E-001 | 0.506 |  | 0.013 | 0.068 | 0.192 | 8.47E-001 | 0.904 |
| **Inferior longitudinal fasciculus** | -0.049 | 0.056 | -0.866 | 3.87E-001 | 0.490 |  | -0.033 | 0.069 | -0.472 | 6.37E-001 | 0.807 |
| **Medial lemniscus** | -0.083 | 0.054 | -1.537 | 1.24E-001 | 0.376 |  | -0.063 | 0.068 | -0.922 | 3.57E-001 | 0.807 |
| **Parahippocampal part of cingulum** | 0.014 | 0.053 | 0.258 | 7.96E-001 | 0.796 |  | 0.047 | 0.062 | 0.767 | 4.43E-001 | 0.807 |
| **Posterior thalamic radiation** | -0.046 | 0.054 | -0.845 | 3.98E-001 | 0.490 |  | -0.044 | 0.067 | -0.648 | 5.17E-001 | 0.807 |
| **Superior longitudinal fasciculus (bilateral)** | -0.066 | 0.056 | -1.188 | 2.35E-001 | 0.376 |  | -0.039 | 0.069 | -0.569 | 5.69E-001 | 0.807 |
| **Superior longitudinal fasciculus (left)** | -0.107 | 0.058 | -1.836 | 6.66E-002 | 0.355 |  | -0.092 | 0.071 | -1.302 | 1.93E-001 | 0.807 |
| **Superior thalamic radiation** | -0.148 | 0.056 | -2.656 | 8.01E-003 | 0.089 |  | -0.079 | 0.070 | -1.122 | 2.62E-001 | 0.807 |
| **Uncinate fasciculus** | -0.064 | 0.053 | -1.195 | 2.32E-001 | 0.376 |  | -0.029 | 0.064 | -0.447 | 6.55E-001 | 0.807 |
| **Forceps major** | -0.151 | 0.059 | -2.544 | 1.11E-002 | 0.089 |  | -0.086 | 0.072 | -1.197 | 2.31E-001 | 0.807 |
| **Forceps minor** | -0.070 | 0.057 | -1.226 | 2.21E-001 | 0.376 |  | -0.066 | 0.072 | -0.922 | 3.57E-001 | 0.807 |
| **Middle cerebellar peduncle** | -0.079 | 0.058 | -1.364 | 1.73E-001 | 0.376 |  | 0.034 | 0.067 | 0.501 | 6.16E-001 | 0.807 |

Table S8. The interaction between MDD definition and hemisphere on FA values of DTI tracts. The results below were for follow-up model to test whether there was a lateralised effect of MDD definition (see main text, section Methods-Statistical methods-White matter integrity). Forceps major and minor and middle cerebellar peduncle were not included in this analysis as they were unilateral tracts. A significant effect of the interaction between recurrent definition and hemisphere was found in superior longitudinal fasciculus, therefore individual tests on the FA values on each hemisphere of superior longitudinal fasciculus was conducted. As the effect of MDD definitions were significant on left superior longitudinal fasciculus, the results were added in Table 1, S6 and S7.

| DTI tracts | **Principal definition** | | | |  |  | **Recurrent definition** | | | |  |
| --- | --- | --- | --- | --- | --- | --- | --- | --- | --- | --- | --- |
|  | Effect size | Standard deviation | t value | p value | p_corrected_ |  | Effect size | Standard deviation | t value | p value | p_corrected_ |
| **Acoustic radiation** | -0.108 | 0.063 | -1.712 | 8.72E-002 | 0.348 |  | -0.069 | 0.067 | -1.034 | 3.01E-001 | 0.932 |
| **Anterior thalamic radiation** | -0.061 | 0.038 | -1.631 | 1.03E-001 | 0.348 |  | -0.026 | 0.041 | -0.631 | 5.28E-001 | 0.932 |
| **Cingulate gyrus part of cingulum** | -0.026 | 0.063 | -0.411 | 6.81E-001 | 0.894 |  | -0.002 | 0.067 | -0.036 | 9.71E-001 | 0.971 |
| **Corticospinal tract** | -0.088 | 0.060 | -1.459 | 1.45E-001 | 0.348 |  | -0.033 | 0.066 | -0.494 | 6.21E-001 | 0.932 |
| **Inferior fronto-occipital fasciculus** | 0.005 | 0.047 | 0.110 | 9.12E-001 | 0.933 |  | 0.038 | 0.050 | 0.753 | 4.51E-001 | 0.932 |
| **Inferior longitudinal fasciculus** | 0.012 | 0.038 | 0.325 | 7.45E-001 | 0.894 |  | 0.024 | 0.041 | 0.582 | 5.61E-001 | 0.932 |
| **Medial lemniscus** | 0.021 | 0.036 | 0.579 | 5.63E-001 | 0.894 |  | 0.031 | 0.039 | 0.801 | 4.23E-001 | 0.932 |
| **Parahippocampal part of cingulum** | -0.044 | 0.071 | -0.628 | 5.30E-001 | 0.894 |  | -0.008 | 0.075 | -0.111 | 9.12E-001 | 0.971 |
| **Posterior thalamic radiation** | -0.071 | 0.047 | -1.517 | 1.30E-001 | 0.348 |  | -0.008 | 0.050 | -0.151 | 8.80E-001 | 0.971 |
| **Superior longitudinal fasciculus** | 0.069 | 0.036 | 1.939 | 5.27E-002 | 0.348 |  | 0.117 | 0.038 | 3.076 | 2.14E-003 | 0.026 |
| **Superior thalamic radiation** | -0.003 | 0.036 | -0.085 | 9.33E-001 | 0.933 |  | 0.023 | 0.038 | 0.596 | 5.51E-001 | 0.932 |
| **Uncinate fasciculus** | 0.021 | 0.057 | 0.369 | 7.12E-001 | 0.894 |  | -0.005 | 0.062 | -0.075 | 9.40E-001 | 0.971 |

Table S9. The effect of MDD definition on FA values of DTI tracts (education level and release included as covariates). There was no significant effect of education level and number of release on the definitions of MDD (see supplementary materials, Methods-MDD definitions). However, in order to double check whether the results would remain the same when these factors were included, a validation test was conducted. The regions that were found significant in the section of results in the main text remained significant (left superior longitudinal fasciculus, forceps major and superior thalamic radiation).

| DTI tracts | **Principal definition** | | | |  |  | **Recurrent definition** | | | |  |
| --- | --- | --- | --- | --- | --- | --- | --- | --- | --- | --- | --- |
|  | Effect size | Standard deviation | t value | p value | p_corrected_ |  | Effect size | Standard deviation | t value | p value | p_corrected_ |
| **Acoustic radiation** | -0.080 | 0.058 | -1.371 | 1.71E-001 | 0.227 |  | -0.089 | 0.062 | -1.428 | 1.53E-001 | 0.239 |
| **Anterior thalamic radiation** | -0.075 | 0.063 | -1.195 | 2.32E-001 | 0.266 |  | -0.062 | 0.067 | -0.926 | 3.55E-001 | 0.464 |
| **Cingulate gyrus part of cingulum** | -0.130 | 0.059 | -2.198 | 2.82E-002 | 0.090 |  | -0.100 | 0.063 | -1.575 | 1.15E-001 | 0.205 |
| **Corticospinal tract** | -0.079 | 0.058 | -1.351 | 1.77E-001 | 0.227 |  | -0.052 | 0.062 | -0.832 | 4.06E-001 | 0.464 |
| **Inferior fronto-occipital fasciculus** | -0.088 | 0.060 | -1.457 | 1.45E-001 | 0.227 |  | -0.055 | 0.065 | -0.847 | 3.97E-001 | 0.464 |
| **Inferior longitudinal fasciculus** | -0.120 | 0.062 | -1.959 | 5.04E-002 | 0.115 |  | -0.121 | 0.065 | -1.849 | 6.47E-002 | 0.171 |
| **Medial lemniscus** | -0.131 | 0.062 | -2.127 | 3.36E-002 | 0.090 |  | -0.138 | 0.065 | -2.119 | 3.43E-002 | 0.110 |
| **Parahippocampal part of cingulum** | -0.040 | 0.058 | -0.696 | 4.87E-001 | 0.487 |  | -0.019 | 0.060 | -0.315 | 7.53E-001 | 0.753 |
| **Posterior thalamic radiation** | -0.081 | 0.061 | -1.327 | 1.85E-001 | 0.227 |  | -0.090 | 0.065 | -1.392 | 1.64E-001 | 0.239 |
| **Superior longitudinal fasciculus (bilateral)** | -0.140 | 0.063 | -2.220 | 2.66E-002 | 0.090 |  | -0.147 | 0.067 | -2.185 | 2.91E-002 | 0.110 |
| **Superior longitudinal fasciculus (left)** | -0.192 | 0.066 | -2.927 | 3.49E-003 | 0.027 |  | -0.217 | 0.069 | -3.127 | 1.80E-003 | 0.029 |
| **Superior thalamic radiation** | -0.225 | 0.065 | -3.464 | 5.53E-004 | 0.009 |  | -0.178 | 0.069 | -2.571 | 1.02E-002 | 0.082 |
| **Uncinate fasciculus** | -0.104 | 0.058 | -1.787 | 7.43E-002 | 0.149 |  | -0.104 | 0.062 | -1.679 | 9.33E-002 | 0.187 |
| **Forceps major** | -0.188 | 0.067 | -2.809 | 5.06E-003 | 0.027 |  | -0.127 | 0.071 | -1.784 | 7.47E-002 | 0.171 |
| **Forceps minor** | -0.111 | 0.065 | -1.702 | 8.91E-002 | 0.158 |  | -0.156 | 0.070 | -2.233 | 2.57E-002 | 0.110 |
| **Middle cerebellar peduncle** | -0.070 | 0.064 | -1.093 | 2.75E-001 | 0.293 |  | 0.035 | 0.068 | 0.518 | 6.05E-001 | 0.645 |

Table S10. Loadings of first latent factor of PCA on global FA, association/commissural fibres, thalamic radiations and projection fibres. The individual tracts included for these four PCA were stated in the main text (Methods-Statistical methods-White matter integrity). All the PCA were performed on the overall sample after outliers were excluded to maximize the accuracy of the models in the largest sample possible (N=4594).

| **Tracts** | **PCA analyses** | | | |
| --- | --- | --- | --- | --- |
|  | **Global FA** | **Association/**  **Commissural fibres** | **Thalamic radiations** | **Projection fibres** |
| Cingulate gyrus part of cingulum (left) | 0.583 | 0.663 | -- | -- |
| Cingulate gyrus part of cingulum (right) | 0.544 | 0.629 | -- | -- |
| Inferior fronto-occipital fasciculus (left) | 0.817 | 0.820 | -- | -- |
| Inferior fronto-occipital fasciculus (right) | 0.836 | 0.824 | -- | -- |
| Inferior longitudinal fasciculus (left) | 0.808 | 0.798 | -- | -- |
| Inferior longitudinal fasciculus (right) | 0.839 | 0.815 | -- | -- |
| Parahippocampal part of cingulum (left) | 0.408 | 0.415 | -- | -- |
| Parahippocampal part of cingulum (right) | 0.356 | 0.356 | -- | -- |
| Superior longitudinal fasciculus (left) | 0.798 | 0.788 | -- | -- |
| Superior longitudinal fasciculus (right) | 0.820 | 0.796 | -- | -- |
| Uncinate fasciculus (left) | 0.657 | 0.678 | -- | -- |
| Uncinate fasciculus (right) | 0.673 | 0.687 | -- | -- |
| Forceps major | 0.539 | 0.551 | -- | -- |
| Forceps minor | 0.784 | 0.782 | -- | -- |
| Anterior thalamic radiation (left) | 0.762 | -- | 0.784 | -- |
| Anterior thalamic radiation (right) | 0.759 | -- | 0.809 | -- |
| Posterior thalamic radiation (left) | 0.645 | -- | 0.761 | -- |
| Posterior thalamic radiation (right) | 0.641 | -- | 0.794 | -- |
| Superior thalamic radiation (left) | 0.636 | -- | 0.744 | -- |
| Superior thalamic radiation (right) | 0.610 | -- | 0.744 | -- |
| Acoustic radiation (left) | 0.607 | -- | -- | 0.536 |
| Acoustic radiation (right) | 0.626 | -- | -- | 0.610 |
| Corticospinal tract (left) | 0.554 | -- | -- | 0.782 |
| Corticospinal tract (right) | 0.552 | -- | -- | 0.800 |
| Medial lemniscus (left) | 0.237 | -- | -- | 0.475 |
| Medial lemniscus (right) | 0.232 | -- | -- | 0.490 |
| Middle cerebellar peduncle | 0.325 | -- | -- | 0.571 |


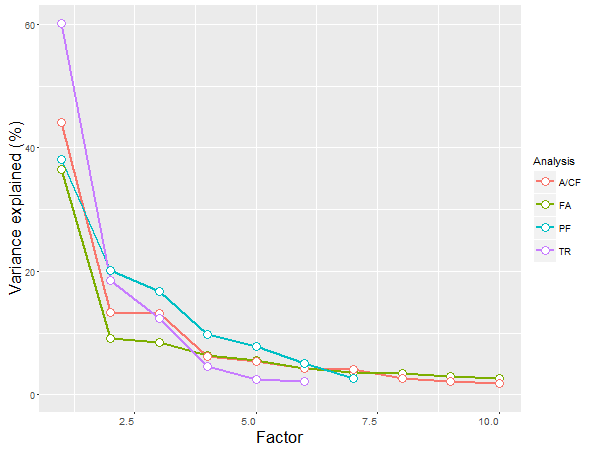


Figure S5. Scree plot of the four PCA analyses on global FA (FA), association/commissural fibres (A/CF), thalamic radiations (TR) and projection fibres (PF). Variance explained were stated in the results of the main text.
